# Loss of GNE Predicts Lymph Node Metastasis in Early Gastric Cancer

**DOI:** 10.3390/cells11223624

**Published:** 2022-11-16

**Authors:** Xinying Guo, Jie Gu, Anwei Xue, Shushu Song, Bo Liu, Xiaodong Gao, Jianxin Gu, Lei Chang, Yuanyuan Ruan

**Affiliations:** 1 NHC Key Laboratory of Glycoconjugates Research, Department of Biochemistry and Molecular Biology, School of Basic Medical Sciences, Fudan University, Shanghai 200032, China; 2 Department of General Surgery, Zhongshan Hospital, Fudan University, Shanghai 200032, China; 3 State Key Laboratory of Proteomics, Beijing Proteome Research Center, National Center for Protein Sciences (Beijing), Beijing Institute of Lifeomics, Beijing 102206, China

**Keywords:** GNE, early gastric cancer, lymph node metastasis, risk factor

## Abstract

Endoscopic surgery is increasingly utilized for the treatment of early gastric cancer (EGC) worldwide, whereas lymph node metastasis (LNM) remains a critical risk factor for the relapse of EGC after endoscopic surgery. Therefore, identifying potential predictive factors and understanding the molecular mechanisms are urgently needed for improving the outcome of EGC patients with LNM. UDP-N-acetylglucosamine 2-epimerase/N-acetylmannosamine kinase (GNE) is the key enzyme in the process of biosynthesis of CMP-Neu5Ac from UDP-N-acetylglucosamine (UDP-GlcNAc), which acts as a substrate for several reactions in glycan metabolism. In this study, we found that GNE was down-regulated in EGC patients with LNM. GNE expression as well as localization, tumor size, intravascular tumor thrombi and Lauren’s classification were further identified as independent predictive factors for LNM. Combining GNE expression with traditional risk factors, including tumor size and differentiation degrees, could generate a better model for predicting LNM in EGC patients. Overall, our study implies that low GNE expression is a potential predictor of LNM in EGC.

## 1. Introduction

Gastric cancer ranks as the fifth-most-frequent malignant tumor and is the fourth-leading cause of cancer-related death worldwide [1]. With the improvement in screening and diagnostic methods, increasing gastric cancer cases are diagnosed at an early stage. Early gastric cancer (EGC) is referred to as invasion confined to mucosa and submucosa, regardless of lymph node metastasis (LNM). According to Japanese Gastric Cancer Association (JGCA) classification guidelines, endoscopic treatment, including endoscopic mucosal resection (EMR) and endoscopic submucosal dissection (ESD), can be utilized to treat EGC patients with a negligible risk of LNM [2]. In general, the incidence of LNM in patients with EGC is around 10%, which could increase the risk of tumor recurrence and lead to endoscopic resection failure [3]. Therefore, searching for possible predictive factors for lymph node metastasis is crucial to deciding the performance of endoscopic mucosal resection or endoscopic submucosal dissection surgery in EGC. Previous studies have focused on pre-/post-operative clinicopathological factors, tumor microenvironment and imaging techniques to predict LNM but reached no standard principle due to low specificity and sensitivity [4]. Recently, biological markers have risen to show effective efforts in lymph node metastasis prediction in early gastric cancer [5]. Nevertheless, there are no consolidated and upgraded risk factors to form a unified prediction pattern. Recommendations of a two-step method were raised, in which suitable patients could undergo endoscopic resection based on endoscopic and histopathologic confirmations firstly and additional surgical intervention could be performed after determining EMR/ESD specimens [6].

Aberrant protein glycosylation is commonly observed in numerous cancers, not only directly impacting cell growth and survival but also facilitating tumor-induced immunomodulation and eventual metastasis [7,8]. Glycosylation, as one of the structurally various and complex forms of post-translational modifications (PTMs) on proteins, has diverse glycoforms, such as O-glycans, N-glycans and glycosaminoglycans [9]. Different forms of glycosylation have distinct linkages between protein and sugar. For example, N-glycosylation occurs on asparagine residues, while O-glycosylation means the transfer of sugars or glycans to serine, threonine or hydroxylysine residues [10]. The main sugar chain donors of human protein glycosylation, such as glucose, galactose, N-acetylglucosamine (GlcNAc), N-acetylgalactosamine (GalNAc), fucose, mannose and 5-mer N-acetylneuraminic acid (Neu5Ac, sialic acid), all might come from glucose metabolism [7]. A minor branch (2–5%) of the glycolytic pathway shunts to the hexosamine biosynthesis pathway (HBP) and generates the final product UDP-N-acetylglucosamine (UDP-GlcNAc) [11]. In addition to direct utilization for protein glycosylation, especially O-GlcNAcylation, UDP-GlcNAc is converted into other glycosyl donors, including CMP-N-acetylneuraminic acid (CMP-Neu5Ac). The synthesis of CMP-Neu5Ac from UDP-GlcNAc requires five enzymatic steps and UDP-N-acetylglucosamine 2-epimerase/N-acetylmannosamine kinase (GNE) catalyzes the first two steps and functions as the key rate-limiting enzyme for CMP-Neu5Ac biosynthesis in vertebrates [12]. The generation of CMP-Neu5Ac contributes to protein sialylation, a common terminal glycan modification involved in the regulation of cell–cell recognition, cell adhesion, antigenicity, protein targeting and invasion [13,14]. Biallelic GNE mutations underlie GNE myopathy, an adult-onset progressive myopathy [15]. However, the role of GNE in cancer progression remains largely unclear. Herein, we examined the clinical significance of GNE in predicting the LNM of EGC and provided a new possible biomarker for the diagnosis of LNM in EGC patients.

## 2. Materials and Methods

### 2.1. Patients and Specimens

In total, 226 early gastric cancer cases with T1a or T1b stages were enrolled in this study. All early gastric cancer patients met the criteria and had undergone standard endoscopic resection without preoperative treatment from Zhongshan hospital (Fudan University, Shanghai, China) from 2013 to 2020. Another independent group of 41 tumor tissues from early gastric cancer patients was also collected from Zhongshan hospital in 2020. The diagnosis of gastric cancer was confirmed by pathologic examination. The patients’ characteristics including gender, age, tumor size, location, Lauren’s classification, differentiation, intravascular tumor thrombi and TNM stage were obtained from medical records. The use of human tissue samples and clinical data was approved by the ethics committee of Zhongshan hospital (B2022-261R). Informed consent was obtained from enrolled patients with awareness of the aim of the study.

### 2.2. Tissue Microarray (TMA) and Immunohistochemistry (IHC)

Briefly, core tissue sections (size around 1.0 mm) were paraffin embedded from the center of early gastric tumor focus and placed in order on slides. The slides were baked at 60 °C for 6–8 h, then dealkylated with xylene, rehydrated in gradient ethanol and blocked with 3% hydrogen peroxide. UltraVision Protein Block (Thermo scientific) was used for non-specific background staining. The sections were immersed in citric acid buffer and microwave oven for antigen extraction. The sections were incubated with GNE (1:100, Proteintech, Cat No. 25079-1-AP) or LYVE-1 (1:100, Proteintech, Cat No. 51011-1-AP) antibody at 4 °C. After washing, the tissue sections were treated with reagents according to manufacturer’s protocol. Finally, the tissue sections were stained with hematoxylin, dehydrated and covered with cover glass for scanning through a computer image system. The percentage and intensity of staining cells were detected by immunohistochemistry. The percentage of immunopositive cells (A) was divided into five grades: 0% (0), 0–25% (1), 25–50% (2), 50–75% (3) and 75–100% (4). The staining intensity (B) was categorized as: negative (0), weak (1), medium (2) and strong (3). The total score of each section was obtained by A × B. The value of the closest point (0, 1) on the curve maximized the sensitivity and specificity of LNM, which was defined as the cut-off score by ROC analysis.

### 2.3. Reverse Transcription PCR and Quantitative Real-Time PCR

RNA samples from fresh tumor tissues were extracted with TRIzol reagent (TAKARA). First-strand cDNAs were synthesized. Relative RNA levels were detected by qRT-PCR using SYBR green on Real-Time PCR System (Applied Biosystems, USA) and calculated using β-actin as internal reference through the comparative CT (2^−ΔΔCT^) method. Sequences of qPCR primers are as follows: *ACTB* (Forward: CACCATTGGCAATGAGCGGTTC; Reverse: AGGTCTTTGCGGATGTCCACGT); *GNE* (Forward: GAAGCATACGCCTCTGGAATGG; Reverse: CAGCCTCATCTTTTGGCACTGAC).

### 2.4. Statistical Analysis

GraphPad Prism 9 (GraphPad, San Diego, CA, USA) and IBM SPSS Version 26 were utilized to conduct statistical analyses. All results were shown as mean ± SD. Student’s two-tailed *t* test was used for comparison between groups. Categorical data were analyzed using Pearson chi-square tests. Logistic regression was employed for univariate analysis of clinicopathological factors for LNM. ROC analysis was used to determine the sensitivity and specificity for predicting LNM by the parameters. Forest plot, nomogram and calibration plot were created with RStudio using ‘ggplot2’ package to establish and verify a new prediction model. All statistical analyses were two-sided and *p* < 0.05 was considered statistically significant.

## 3. Results

### 3.1. GNE Is Down-Regulated in EGCs with Lymph Node Metastasis

**Figure 1:**
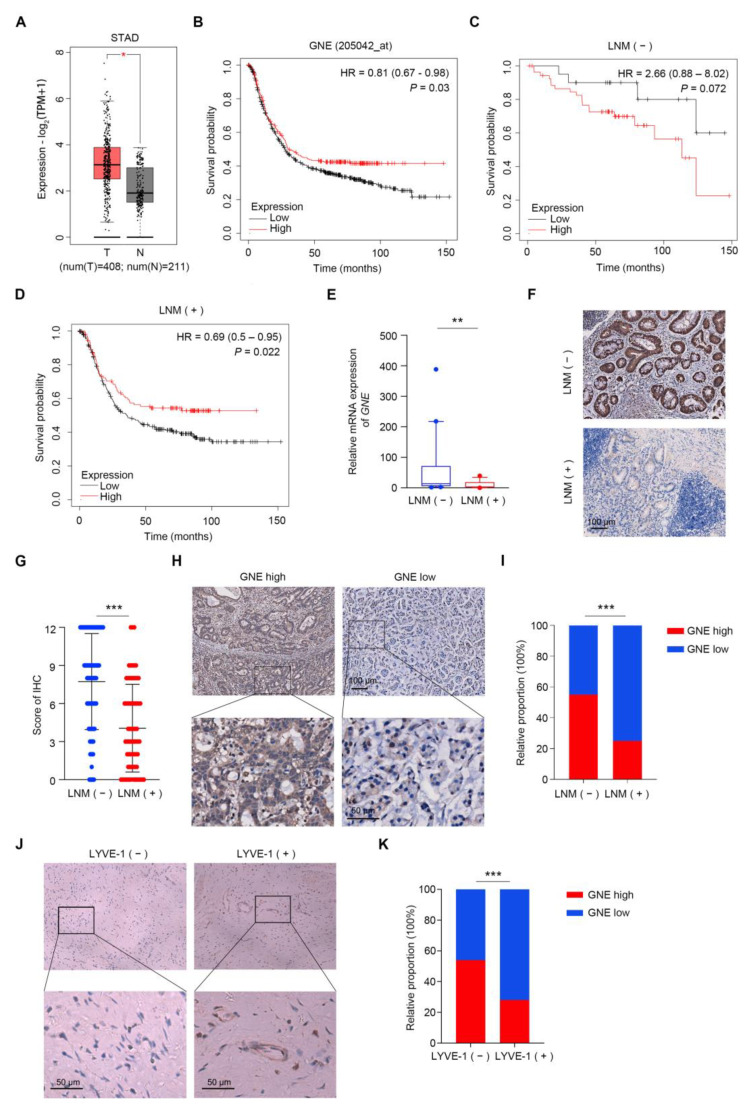
GNE is down-regulated in EGCs with lymph node metastasis. (**A**) Results of analysis of GNE expression level in tumor (T) and normal (N) of TCGA_STAD dataset from GEPIA database; (**B**–**D**) correlation of GNE with the survival of gastric cancer patients was analyzed utilizing an online survival analysis software (https://kmplot.com/analysis/index.php?p=service&cancer=gastric (accessed on 5 May 2022)); (**B**) all gastric cancer patients; (**C**) group of gastric cancer patients without LNM; (**D**) group of gastric cancer patients with LNM; (**E**) the mRNA levels of GNE were evaluated in fresh EGC tissues with (*n* = 16) or without (*n* = 25) LNM; (**F**,**G**) GNE expression was assessed by immunohistochemistry in TMA containing EGC specimens with or without LNM. Representative images are shown in (**F**); (**H**) representative images of GNE low- and high-expression tissues; (**I**) the classification of patients with high or low expression of GNE in LNM (−) and LNM (+) groups in percentage terms; (**J**) representative images of LYVE-1 (−) and LYVE-1 (+) tissues; (**K**) the classification of patients with high or low expression of GNE in LYVE-1 (−) and LYVE-1 (+) groups in percentage terms. *, *p* < 0.05; **, *p* < 0.01; ***, *p* < 0.001.

In order to investigate the differential expression of GNE in gastric cancer, we analyzed the TCGA_STAD dataset (Stomach Adenocarcinoma dataset from The Cancer Genome Atlas) based on the GEPIA database and the results showed that the expression of GNE increased significantly in the tumor group (Figure 1A). Next, we explored the prognostic significance of GNE expression, utilizing an online survival analysis software (https://kmplot.com/analysis/index.php?p=service&cancer=gastric (accessed on 5 May 2022). Results indicated that a low expression of GNE was remarkably associated with poor prognosis in gastric cancer patients (Figure 1B). We further analyzed prognostic significance of GNE expression among groups with or without LNM and found that the significant correlation between a low expression of GNE and poor prognosis existed only in gastric cancer patients with LNM, suggesting that the prognostic value of GNE might be dependent on the LNM status of gastric cancer patients (Figure 1C,D). Next, we performed real-time PCR analysis to examine the GNE expression in early gastric cancer clinical samples with or without LNM. The results indicated that the mRNA levels of GNE were apparently lower in metastatic EGCs compared to that in non-metastatic ones (Figure 1E). To further confirm the change in GNE expression, tissue microarrays of 226 EGC cases were performed using an immunohistochemistry (IHC) assay. The results showed that early gastric cancer cases with LNM presented lower GNE protein expression levels in comparison with those without LNM, which was consistent with the result of real-time PCR (Figure 1F,G). Moreover, the distribution of GNE in observation was mainly within the cytoplasm of the tumor cells (Figure 1F). Based on the receiver operating characteristic (ROC) curve analysis, GNE IHC staining scores of less than 7 were considered as low expression. Figure 1H displays representative images of low and high expressions of GNE. Statistical analysis indicated a significant correlation between a low expression of GNE and LNM in early gastric cancer (*p* < 0.001) (Figure 1I). To estimate the extent of lymphatic vessel invasion in the above 226 EGC cases, we also conducted IHC with an antibody against LYVE-1. Representative images of LYVE-1 (−) and LYVE-1 (+) are shown in Figure 1J. We found that the proportion of low expressions of GNE in the LYVE (+) group was higher than that in the LYVE (−) group (Figure 1K), which was consistent with our previous analysis. These results suggested that GNE was down-regulated in EGC patients with lymph node metastasis.

### 3.2. Correlation between GNE Expression and Clinicopathological Features in EGC Patients

In order to further explore the association between GNE expression and various clinical characteristics of EGC patients, Pearson chi-square tests based on the information of the aforementioned 226 early gastric cancer patients were performed. The analysis indicated that a low expression of GNE was significantly correlated with Lauren’s classification (*p* = 0.013), poor differentiation (*p* = 0.025) and LNM (*p* < 0.001) (Table 1).

### 3.3. Identification of Risk Factors for Lymph Node Metastasis in Early Gastric Cancer

To elucidate the linkages between LNM and various clinicopathological factors, we analyzed all T1 patients as well as the T1a and T1b subgroups separately. Lymph node metastasis in all T1 patients was significantly correlated with distal localization (*p* = 0.009), larger size (*p* = 0.011), intravascular tumor thrombi (*p* < 0.001), diffuse/mixed Lauren’s classification (*p* < 0.001) and GNE expression (*p* < 0.001) (Table 2). In T1a subgroups, only intravascular tumor thrombi (*p* < 0.001) and GNE expression (*p* = 0.018) were associated with LNM, probably due to the limited sample size (Table 2). In T1b subgroups, similar LNM-related clinicopathological factors were identified as those for all T1 patients (Table 2).

Next, univariate logistic regression analysis was applied to identify dependent clinicopathological factors associated with lymph node metastasis in early gastric cancer. Localization (odds ratio (OR), 2.577; 95% confidence interval (CI), 1.246–5.328; *p* = 0.011), diameter (OR, 2.088; 95% CI, 1.175–3.710; *p* = 0.012), intravascular tumor thrombi (OR, 5.400; 95% CI, 2.644–11.027; *p* < 0.001), Lauren’s classification (OR, 3.056; 95% CI, 1.688–5.532; *p* = 0.001) and GNE expression (OR, 0.262; 95% CI, 0.141–0.487; *p* < 0.001) were identified as independent risk factors that affect LNM in EGC (Table 3).

### 3.4. ROC Curve and Nomogram Model for Predicting Lymph Node Metastasis in Early Gastric Cancer

**Figure 2:**
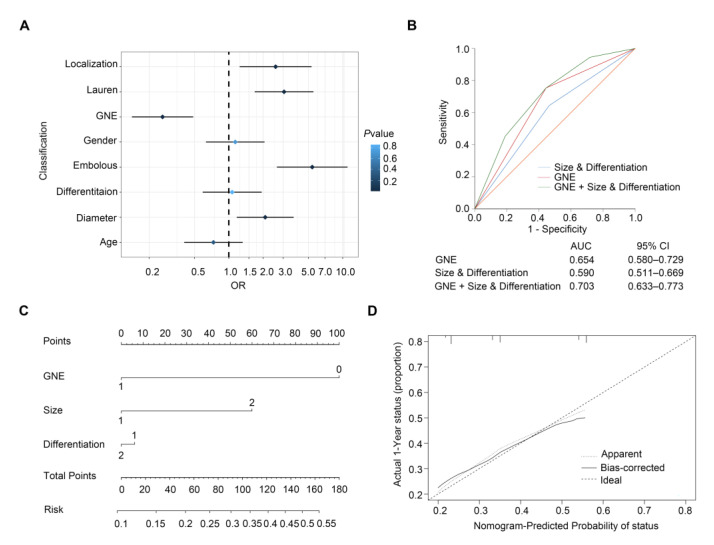
Predictive models for lymph node metastasis in early gastric cancer. (**A**) Forest plot showing the results of univariable logistic regression analysis for LNM in EGC; (**B**) ROC analysis of predictive value for combined size and differentiation with GNE model, size and differentiation model and GNE model in LNM of early gastric cancer patients; (**C**) nomogram generation for prediction of LNM integrating GNE expression (0 and 1 represent low expression and high expression), tumor size (1 and 2 represent ≤2 cm and >2 cm) and differentiation (1 and 2 represent poor differentiation and good and moderate differentiation); (**D**) calibration curve for nomogram-predicted probability of LNM.

We established a forest plot to better illustrate the results of the analysis of risk factors for lymph node metastasis in early gastric cancer (Figure 2A). The traditional model for predicting lymph node metastasis of EGC is based on clinicopathological variables of tumor size and differentiation. In order to assess the ascendency of our newly built model over the traditional model based on the combination of size and differentiation, we compared the predictive value of the traditional model with that of incorporating GNE expression with traditional factors. Results of the ROC curve showed that AUC value of GNE, size and differentiation (AUC (95% CI), 0.703 (0.633–0.773)) was much higher than that of clinicopathological variables alone (AUC (95% CI), 0.590 (0.511–0.669)) (*p* < 0.001) or GNE alone (AUC (95% CI), 0.654 (0.580–0.729)) (*p* < 0.001) (Figure 2B). This demonstrated that GNE protein level was rather meaningful to LNM. Furthermore, a nomogram comprising GNE expression, size and differentiation was constructed to describe a quantification for better prediction of LNM in EGC, in which a higher risk value represents a higher occurrence of LNM (Figure 2C). The calibration plot illustrated a good performance of the nomogram with the ideal stimulation model (Figure 2D). In summary, our model collaborating GNE expression and clinicopathological features proved that incorporating GNE could help to achieve superior prediction for lymph node metastasis in early gastric cancer.

## 4. Discussion

Conventional gastrectomy was the gold standard for EGC, owing to good prognosis [16] However, D2 resection surgery increased mortality and morbidity rates and influenced the occurrence of surgical complications and patients’ quality of life [17]. Endoscopic resection excelled in minimal invasion, all-stomach conservation and postoperative quality of life when patients met standard criteria with tumor appearance and size [18]. However, LNM occurrence could cause endoscopic resection failure, thus, ruling out that LNM was vital to EGC patients. The guidelines for gastric cancer from the National Comprehensive Cancer Network (NCCN) recommend gastroscopy, computed tomography (CT) or endoscopic ultrasonography (EUS) to assess lymph node status preoperatively [19], while none of these detections could accurately examine LNM alone. Postoperative factors, such as tumor size, differentiation, ulceration, depth of invasion [20] and collagen signature [21], were brought up to predict LNM in EGC. In addition, lncRNA [22], miRNA [23] and protein biomarkers [24,25,26] were shown to correlate to LNM in EGC, yet no concordant prediction model was established using the above markers.

In this study, we, for the first time, discovered the predictive feature of GNE for lymph node metastasis in early gastric cancer using a retrospective study. Since the odds for LNM are low and LNM is hard to notice without recurrence and further tests, the tissue microarray employed in this study appears to be particularly valuable in that it contains LNM status and detailed information of clinical and pathological characteristics of enrolled early gastric cancer patients. However, there are limitations, including that the number of patient cases is restricted and the LNM status merely represents the status at the information recorded time, for which the future progression of gastric cancer remains unsuspected. Meanwhile, further study should be focused on the mechanism of how low expressions of GNE regulate metastasis and the contradiction of hypersialylation mostly correlated with metastasis in cancers. A possible explanation is that hypersialytion and GNE have negative feedback that causes reductions in GNE expression while the substrate of GNE may be accumulated with low expressions of GNE. Nevertheless, the level of hypersialylation was not detected due to the complexity and heterogeneity of fresh tumor samples in the study.

N-linked and O-linked glycans are constructed by various sugars, among which the sialic acids at the end of glycans are of great importance [27]. N-acetylneuraminic acid (Neu5Ac), as the most common sialic acid in humans, participates in different kinds of cellular interactions, including interactions with extracellular matrix (ECM), epithelial cells and immune cells [28]. The biosynthesis of sialylated glycans needs sialytransferase enzymes, which are Golgi resident and membrane bound. There are 20 subtypes of sialytrasferase enzymes in humans, all of which use CMP-Neu5Ac as the donor [29]. The starting compound for sialic acid biosynthesis is the product of HBP and UDP-GlcNAc and the synthesis of CMP-Neu5Ac from UDP-GlcNAc requires five enzymatic steps [30]. GNE is the key enzyme in the process of CMP-Neu5Ac synthesis and has been proved as a master regulator for sialylations [31]. Hypersialylation of around 40–60% of tumor cell surfaces is found to be the hallmark of some kinds of cancers, such as pancreatic cancer [32], breast cancer [33] and lung cancer [34]. One of the critical processes in tumor metastasis is reduced adhesion between cancer cells and ECM or other adjacent cells, which allows cancer cells to separate and invade much more easily. Sialylation of molecules related to adhesion, such as integrins, could increase the migratory and invasive ability of cancer cells and lead to more metastasis sites [35]. Sialylation of integrins affects integrin cell functions to a large extent. Previous research reported that sialylation promoted integrin-mediated cell mobility on collagen and fibronectin in pancreatic cancer [36]. Selectins are a kind of cell-adhesion receptor, binding sialylated glycans and involved in many adhesion processes, which also plays an important role in cancer metastasis. Elevated α-2,3 sialylation was promoted by ST3Gal IV expression, which increased the metastatic feature in gastric cancer [37]. The phenomenon that global inhibition of sialylation promoted EMT in a mutual affected way was found in HaCaT keratinocytes [38]. However, as the rate-limiting enzyme in the synthesis of CMP-Neu5Ac, GNE has been found to be down-regulated in several kinds of cancers. One study showed that Morris hepatoma exhibited less than 10% of the GNE activity of the normal liver [39]. In addition, it was revealed that methylation of the GNE promoter triggers a decrease in GNE transcription levels in cancers [40] and in HIV-infected lymphocytes [41].

Our research proposed that GNE as a possible biomarker for LNM prediction in EGC for the expression of GNE was confirmed to be down-regulated in EGC patients with LNM. Furthermore, the established prediction models suggested the predictive value of GNE for LNM, which provided a new idea for the assessment and diagnosis of LNM in EGC.

## Figures and Tables

**Table 1:** Correlation between intratumoral GNE expression and clinical characteristics of early gastric cancer.

Factors		GNE Expression		
		Low	High	
	No.	No.	No.	*p*-value
Gender				0.223
Male	153	79	74	
Female	73	44	29	
Age (years)				0.651
<65	144	80	64	
≥65	82	43	39	
Tumor size (cm)				0.553
≤2	108	61	47	
>2	118	62	56	
Tumor location				0.787
Proximal + Middle	59	33	26	
Distal	167	90	77	
Lauren’s classification				0.013
Intestinal	109	50	59	
Diffuse + Mixed	117	73	44	
Differentiation				0.025
Poorly	72	47	25	
Well + Moderately	154	76	78	
Intravascular tumor thrombi				0.695
No	184	99	85	
Yes	42	24	18	
T classification				0.162
T1a	56	35	21	
T1b	170	88	82	
N classification				<0.001
LN (+)	73	55	18	
LN (−)	153	68	85	

**Table 2:** Correlation of lymph node metastasis with clinicopathological characteristics in EGC.

Factors	All T1 Patients					T1a					T1b				
	Patients		LNM			Patients		LNM			Patients		LNM		
	No.	%	−	+	*p*-Value	No.	%	−	+	*p*-Value	No.	%	−	+	*p*-Value
All patients	226	100	153	73		56	100	48	8		170	100	105	65	
Age					0.302					0.430					0.445
<65	144	63.7	94	50		48	85.7	29	6		105	61.7	65	44	
≥65	82	36.3	59	23		8	14.3	19	2		65	38.3	40	21	
Gender					0.666					0.300					0.905
Female	73	32.3	48	25		19	33.9	15	4		54	31.8	33	21	
Male	153	67.7	105	48		37	66.1	33	4		116	68.2	72	44	
Localization					0.009					0.277					0.019
Proximal + Middle	59	26.1	48	11		16	28.6	15	1		43	25.3	33	10	
Distal	167	73.9	105	62		40	71.4	33	7		127	74.7	72	55	
Diameter					0.011					0.716					0.007
≤2 cm	108	47.8	82	26		28	50	24	4		80	47.1	58	22	
>2 cm	118	52.2	71	47		28	50	23	5		90	52.9	47	43	
Differentiation					0.820					0.640					0.646
Well + Moderately	154	68.1	105	49		38	67.9	32	6		116	68.2	73	43	
Poorly	72	31.9	48	24		18	32.1	16	2		54	31.8	32	22	
Intravascular tumor thrombi					<0.001					<0.001					<0.001
No	184	81.4	138	46		54	96.4	48	6		130	76.5	90	40	
Yes	42	18.6	15	27		2	3.6	0	2		40	23.5	15	25	
Lauren’s classification					<0.001					0.661					<0.001
Intestinal	109	48.2	87	22		31	55.4	26	5		78	45.9	61	17	
Diffuse + Mixed	117	51.8	66	51		25	44.6	22	3		92	54.1	44	48	
GNE expression					<0.001					0.018					<0.001
Low	123	54.4	68	55		35	62.5	27	8		88	51.7	41	47	
High	103	45.6	85	18		21	37.5	21	0		82	48.3	64	18	

**Table 3:** Univariate logistic regression analyses for lymph node metastasis in EGC.

Factors	Univariate	
	OR (95% CI)	*p*-value
Age		0.303
<65	1.00 (reference)	
≥65	0.733 (0.406–1.324)	
Gender		0.666
Male	1.00 (reference)	
Female	1.139 (0.630–2.059)	
Localization		0.011
Proximal + Middle	1.00 (reference)	
Distal	2.577 (1.246–5.328)	
Diameter		0.012
≤2 cm	1.00 (reference)	
>2 cm	2.088 (1.175–3.710)	
Differentiation		0.820
Well + Moderately	1.00 (reference)	
Poorly	1.071 (0.590–1.944)	
Intravascular tumor thrombi		<0.001
No	1.00 (reference)	
Yes	5.400 (2.644–11.027)	
Lauren’s classification		<0.001
Intestinal	1.00 (reference)	
Diffuse + Mixed	3.056 (1.688–5.532)	
GNE expression		<0.001
Low	1.00 (reference)	
High	0.262 (0.141–0.487)	

## Data Availability

Data in the TCGA_STAD dataset were obtained from the GEPIA database. Data on survival analysis were obtained from the software (https://kmplot.com/analysis/index.php?p=service&cancer=gastric (accessed on 5 May 2022). The data presented in this study are available in this article.

## References

[1] Sung H., Ferlay J., Siegel R.L., Laversanne M., Soerjomataram I., Jemal A., Bray F. (2021). Global Cancer Statistics 2020: GLOBOCAN Estimates of Incidence and Mortality Worldwide for 36 Cancers in 185 Countries. CA Cancer J. Clin..

[2] Eom S.S., Choi W., Eom B.W., Park S.H., Kim S.J., Kim Y.I., Yoon H.M., Lee J.Y., Kim C.G., Kim H.K. (2022). A Comprehensive and Comparative Review of Global Gastric Cancer Treatment Guidelines. J. Gastric Cancer.

[3] Tanaka N., Katai H., Taniguchi H., Saka M., Morita S., Fukagawa T., Gotoda T. (2010). Trends in characteristics of surgically treated early gastric cancer patients after the introduction of gastric cancer treatment guidelines in Japan. Gastric Cancer.

[4] Chen D., Chen G., Jiang W., Fu M., Liu W., Sui J., Xu S., Liu Z., Zheng X., Chi L. (2019). Association of the Collagen Signature in the Tumor Microenvironment with Lymph Node Metastasis in Early Gastric Cancer. JAMA Surg..

[5] Fang C., Shi J., Sun Q., Gold J.S., Xu G.F., Liu W.J., Zou X.P., Huang Q. (2016). Risk factors of lymph node metastasis in early gastric carcinomas diagnosed by WHO criteria in 379 Chinese patients. J. Dig. Dis..

[6] Shin N., Jeon T.Y., Kim G.H., Park D.Y. (2014). Unveiling lymph node metastasis in early gastric cancer. World J. Gastroenterol..

[7] Stowell S.R., Ju T., Cummings R.D. (2015). Protein glycosylation in cancer. Annu. Rev. Pathol..

[8] Rodrigues J.G., Duarte H.O., Reis C.A., Gomes J. (2021). Aberrant protein glycosylation in cancer: Implications in targeted therapy. Biochem. Soc. Trans..

[9] Kaur H. (2021). Characterization of glycosylation in monoclonal antibodies and its importance in therapeutic antibody development. Crit. Rev. Biotechnol..

[10] Schjoldager K.T., Narimatsu Y., Joshi H.J., Clausen H. (2020). Global view of human protein glycosylation pathways and functions. Nat. Rev. Mol. Cell Biol..

[11] Marshall S., Bacote V., Traxinger R.R. (1991). Discovery of a metabolic pathway mediating glucose-induced desensitization of the glucose transport system. Role of hexosamine biosynthesis in the induction of insulin resistance. J. Biol. Chem..

[12] Hinderlich S., Weidemann W., Yardeni T., Horstkorte R., Huizing M. (2015). UDP-GlcNAc 2-Epimerase/ManNAc Kinase (GNE): A Master Regulator of Sialic Acid Synthesis. Top Curr. Chem..

[13] Dobie C., Skropeta D. (2021). Insights into the role of sialylation in cancer progression and metastasis. Br. J. Cancer.

[14] Vajaria B.N., Patel K.R., Begum R., Patel P.S. (2016). Sialylation: An Avenue to Target Cancer Cells. Pathol. Oncol. Res..

[15] Celeste F.V., Vilboux T., Ciccone C., de Dios J.K., Malicdan M.C., Leoyklang P., McKew J.C., Gahl W.A., Carrillo-Carrasco N., Huizing M. (2014). Mutation update for GNE gene variants associated with GNE myopathy. Hum. Mutat..

[16] Sano T., Sasako M., Kinoshita T., Kinoshita T., Maruyama K., Maruyama K. (1993). Recurrence of early gastric cancer. Follow-up of 1475 patients and review of the Japanese literature. Cancer.

[17] Sasako M. (1997). Risk factors for surgical treatment in the Dutch Gastric Cancer Trial. Br. J. Surg..

[18] Tsujitani S., Oka S., Saito H., Kondo A., Ikeguchi M., Maeta M., Kaibara N. (1999). Less invasive surgery for early gastric cancer based on the low probability of lymph node metastasis. Surgery.

[19] Qiu H., Zhou Z. (2018). Updates and interpretation on NCCN clinical practice guidelines for gastric cancer 2017 version 5. Chin. J. Gastrointest. Surg..

[20] Gotoda T., Yanagisawa A., Sasako M., Ono H., Nakanishi Y., Shimoda T., Kato Y. (2000). Incidence of lymph node metastasis from early gastric cancer: Estimation with a large number of cases at two large centers. Gastric Cancer.

[21] Li H., Huo Z.B., Kong F.T., He Q.Q., Gao Y.H., Liang W.Q., Liu D.X. (2018). Predictive factors for lymph node metastasis and defining a subgroup treatable for laparoscopic lymph node dissection after endoscopic submucosal dissection in poorly differentiated early gastric cancer. World J. Gastrointest. Oncol..

[22] Hong J.H., Jin E.A.-O., Kang H., Chang I.A., Lee S.A.-O., Sung J.A.-O.X. (2019). Correlations between Genetic Polymorphisms in Long Non-Coding RNA PRNCR1 and Gastric Cancer Risk in a Korean Population. Int. J. Mol. Sci..

[23] Feng R., Lu S., Sah B.K., Beeharry M.K., Zhang H., Yan M., Liu B., Li C., Zhu Z. (2018). Serum miR-126 level combined with multi- detector computed tomography in the preoperative prediction of lymph node metastasis of gastric cancer. Cancer Biomark..

[24] Zhu X., Wang Y., Li H., Xue W., Wang R., Wang L., Zhu M., Zheng L. (2017). Deficiency of hMLH1 and hMSH2 expression is a poor prognostic factor in Early Gastric Cancer (EGC). J. Cancer.

[25] Zhao J., Shu P., Duan F., Wang X., Min L., Shen Z., Ruan Y., Qin J., Sun Y., Qin X. (2016). Loss of OLFM4 promotes tumor migration through inducing interleukin-8 expression and predicts lymph node metastasis in early gastric cancer. Oncogenesis.

[26] Yi Kim D., Kyoon Joo J., Kyu Park Y., Yeob Ryu S., Soo Kim H., Kyun Noh B., Hwa Lee K., Hyuk Lee J. (2007). E-cadherin expression in early gastric carcinoma and correlation with lymph node metastasis. J. Surg. Oncol..

[27] Varki A. (2008). Sialic acids in human health and disease. Trends Mol. Med..

[28] Van de Wall S., Santegoets K.C.M., van Houtum E.J.H., Bull C., Adema G.J. (2020). Sialoglycans and Siglecs Can Shape the Tumor Immune Microenvironment. Trends Immunol..

[29] Li Y., Chen X. (2012). Sialic acid metabolism and sialyltransferases: Natural functions and applications. Appl. Microbiol. Biotechnol..

[30] Luchansky S.J., Yarema K.J., Takahashi S., Bertozzi C.R. (2003). GlcNAc 2-epimerase can serve a catabolic role in sialic acid metabolism. J. Biol. Chem..

[31] Keppler O.T., Hinderlich S., Langner J., Schwartz-Albiez R., Reutter W., Pawlita M. (1999). UDP-GlcNAc 2-epimerase: A regulator of cell surface sialylation. Science.

[32] Rodriguez E., Boelaars K., Brown K., Eveline Li R.J., Kruijssen L., Bruijns S.C.M., van Ee T., Schetters S.T.T., Crommentuijn M.H.W., van der Horst J.C. (2021). Sialic acids in pancreatic cancer cells drive tumour-associated macrophage differentiation via the Siglec receptors Siglec-7 and Siglec-9. Nat. Commun..

[33] Nagasundaram M., Horstkorte R., Gnanapragassam V.S. (2020). Sialic Acid Metabolic Engineering of Breast Cancer Cells Interferes with Adhesion and Migration. Molecules.

[34] Elgohary M.M., Helmy M.W., Abdelfattah E.Z.A., Ragab D.M., Mortada S.M., Fang J.Y., Elzoghby A.O. (2018). Targeting sialic acid residues on lung cancer cells by inhalable boronic acid-decorated albumin nanocomposites for combined chemo/herbal therapy. J. Control. Release.

[35] Liu Y.M., Pan D., Bellis S.L., Song Y.H. (2008). Effect of altered glycosylation on the structure of the I-like domain of beta 1 integrin: A molecular dynamics study. Proteins.

[36] Almaraz R.T., Tian Y., Bhattarcharya R., Tan E., Chen S.H., Dallas M.R., Chen L., Zhang Z., Zhang H., Konstantopoulos K. (2012). Metabolic flux increases glycoprotein sialylation: Implications for cell adhesion and cancer metastasis. Mol. Cell. Proteom..

[37] Shen L., Luo Z., Wu J., Qiu L., Luo M., Ke Q., Dong X. (2017). Enhanced expression of alpha2,3-linked sialic acids promotes gastric cancer cell metastasis and correlates with poor prognosis. Int. J. Oncol..

[38] Du J., Hong S., Dong L., Cheng B., Lin L., Zhao B., Chen Y.G., Chen X. (2015). Dynamic Sialylation in Transforming Growth Factor-beta (TGF-beta)-induced Epithelial to Mesenchymal Transition. J. Biol. Chem..

[39] Harms E., Kreisel W., Morris H.P., Reutter W. (1973). Biosynthesis of N-acetylneuraminic acid in Morris hepatomas. Eur. J. Biochem..

[40] Oetke C., Hinderlich S., Reutter W., Pawlita M. (2003). Epigenetically mediated loss of UDP-GlcNAc 2-epimerase/ManNAc kinase expression in hyposialylated cell lines. Biochem. Biophys. Res. Commun..

[41] Giordanengo V., Ollier L., Lanteri M., Lesimple J., March D., Thyss S., Lefebvre J.C. (2004). Epigenetic reprogramming of UDP-N-acetylglucosamine 2-epimerase/N-acetylmannosamine kinase (GNE) in HIV-1-infected CEM T cells. FASEB J..

